# HTLV-1 Tax upregulates early growth response protein 1 through nuclear factor-κB signaling

**DOI:** 10.18632/oncotarget.17699

**Published:** 2017-05-08

**Authors:** Qingsong Huang, Zhiguo Niu, Jingxian Han, Xihong Liu, Zhuangwei Lv, Huanhuan Li, Lixiang Yuan, Xiangping Li, Shuming Sun, Hui Wang, Xinxiang Huang

**Affiliations:** ^1^ School of Medicine, Jiangsu University, Zhenjiang, Jiangsu, China; ^2^ Research Center for Immunology, School of Laboratory Medicine, Xinxiang Medical University, Xinxiang, Henan, China; ^3^ Collaborative Innovation Center of Molecular Diagnosis and Laboratory Medicine, Xinxiang Medical University, Xinxiang, Henan, China; ^4^ The 7th Hospital of Zhengzhou City, Zhengzhou, Henan, China; ^5^ The Third Affiliated Hospital Of Xinxiang Medical University, Xinxiang Medical University, Xinxiang, Henan, China; ^6^ Luoyang Orthopedic-Traumatological Hospital, Zhengzhou, Henan, China

**Keywords:** HTLV-1, Tax, EGR1, NF-κB, adult T cell leukemia

## Abstract

Human T cell leukemia virus type 1 (HTLV-1) is a complex retrovirus that causes adult T cell leukemia (ATL) in susceptible individuals. The HTLV-1-encoded oncoprotein Tax induces persistent activation of the nuclear factor-κB (NF-κB) pathway. Early growth response protein 1 (EGR1) is overexpressed in HTLV-1-infected T cell lines and ATL cells. Here, we showed that both Tax expression and HTLV-1 infection promoted EGR1 overexpression. Loss of the NF-κB binding site in the EGR1 promotor or inhibition of NF-κB activation reduced Tax-induced EGR1 upregulation. Tax mutants unable to activate NF-κB induced only slight EGR1 upregulation as compared with wild-type Tax, confirming NF-κB pathway involvement in EGR1 regulation. Tax also directly interacted with the EGR1 protein and increased endogenous EGR1 stability. Elevated EGR1 in turn promoted p65 nuclear translocation and increased NF-κB activation. These results demonstrate a positive feedback loop between EGR1 expression and NF-κB activation in HTLV-1-infected and Tax-expressing cells. Both NF-κB activation and Tax-induced EGR1 stability upregulated EGR1, which in turn enhanced constitutive NF-κB activation and facilitated ATL progression in HTLV-1-infected cells. These findings suggest EGR1 may be an effective anti-ATL therapeutic target.

## INTRODUCTION

Adult T-cell leukemia/lymphoma (ATL) is characterized by the malignant proliferation of CD4^+^ T lymphocytes infected with human T cell leukemia virus type 1 (HTLV-1) [1, 2]. HTLV-1 viral proteins, especially Tax, modulate cellular gene expression through the cyclic AMP responsive element binding protein/activating transcription factors (CREB/ATF)-, serum response factor (SRF)- and nuclear factor-κB (NF-κB)-associated pathways [3]. NF-κB activity is tightly controlled and is only transiently elevated upon stimulation in normal T cells, but is constitutively activated in HTLV-1-infected T cells [3]. Tax-mediated constitutive activation of NF-κB signaling is essential for HTLV-1 infection-induced T cell transformation [4]. To study the roles of HTLV-1 Tax in ATL, we stably expressed Tax in Jurkat cells (referred to hereafter as TaxP cells). Genome-wide screens revealed a number of genes highly expressed in TaxP cells, including early growth response factor 1 (EGR1) [5].

EGR1 is an immediate-early response protein that can be rapidly and transiently induced by various stimuli, including growth factors, cytokines, mechanical injury, or shear stress [6]. A subgroup of known EGR1 transcriptional targets, including platelet-derived growth factor (PDGF), insulin-like growth factor-II (IGF-II), and transforming growth factor-β1 (TGF-β1), appear to enhance carcinogenic progression [7, 8]. Moreover, EGR1 regulates cell adhesion to the extracellular matrix through induction of TGF-β1, IL-2, and IL-2R/CD25 expression in human T cells [9], and modulates important biological processes, including neuronal outgrowth [10], wound repair [11, 12], growth control [13, 14], and apoptosis [15, 16]. In addition, EGR1 upregulates the expression of hsa-miR-106a which post-transcriptionally decreases IL-10 mRNA translation [17]. Soluble factors and miRNAs, including miR181a, miR-146a, and miR-675, are reportedly involved in EGR1 expression regulation [15, 18–20]. Five serum response elements (SREs) and two cAMP response elements (CREs) are located in the EGR1 promoter region [6]. HTLV-1 Tax activates expression of immediate early genes that are regulated predominantly by SREs [21], which implies a potential role for Tax in EGR1 regulation. HTLV-1 Tax was previously reported to transcriptionally promote EGR1 expression, although the role of elevated EGR1 in ATL progression remains unknown.

NF-κB p65 subunit-induced transcription of human immunodeficiency virus 1 (HIV) long terminal repeats was previously shown to be dependent on an interaction with the Sp1 zinc finger DNA-binding domain [22]. EGR1, whose DNA-binding domain shares a high degree of homology with that of Sp1, interacts with p65 *in vitro* and regulates NF-κB transcriptional activity *in vivo* [23, 24]. However, the roles played by EGR1 in regulating NF-κB transcriptional activity in HTLV-1 infected cells also remain unclear.

Our previous study revealed that Tax induces EGR1 expression [5]. The present study investigated the mechanisms involved in EGR1 regulation by Tax, and the relationship between EGR1 expression and NF-κB activation.

## RESULTS

### HTLV-1 infection and Tax upregulate EGR1 expression

**Figure 1:**
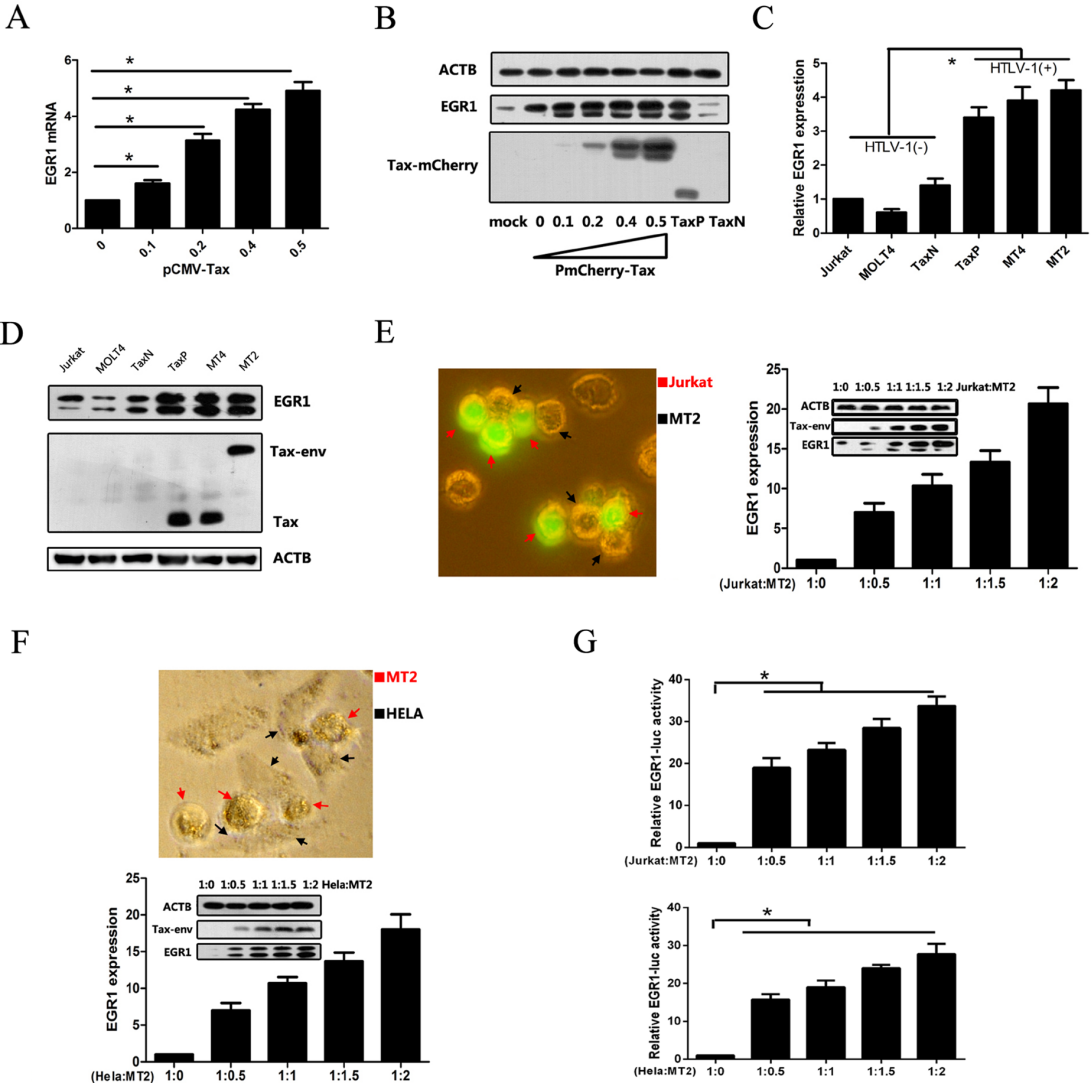
HTLV-1 infection and Tax upregulate EGR1 expression Jurkat cells were transfected with pCMV-Tax plasmids for 48 h, and EGR1 expression was assessed via real-time PCR (**A**) Jurkat cells were transfected with PmCherry-Tax plasmids for 48 h, and EGR1, Tax, and β-Actin (ATCB; loading control) were detected in these, TaxN, and TaxP cells via western blot (**B**) EGR1 mRNA (**C**) and protein (**D**) levels were measured in different cell lines, including Jurkat, MOLT4, TaxN, TaxP, MT4, and MT2. Jurkat cells were labeled with CFSE and cultured with HTLV-1 positive MT2 cells at the indicated final ratios for 48 h (**E**) A representative image shows MT2 cells (black arrows) contacting Jurkat cells (red arrows) after 4 h of co-culture (left panel). CFSE-labeled Jurkat cells were separated by flow cytometry and EGR1 and Tax protein levels were assessed via western blot. EGR1 mRNA in the separated cells was measured using real-time PCR. Hela cells were co-cultured with MT2 cells at the indicated ratios for 48 h and then collected after washing away MT2 cells (**F**) A representative image shows MT2 cells (red arrows) contacting Hela cells (black arrows) after 4 h of co-culture (left panel). EGR1 mRNA and protein levels, and ACTB and Tax protein levels, were detected via real-time PCR or western blot. Jurkat (upper panel) and Hela cells (bottom panel) were transfected with EGR-luc plasmids for 24 h, washed with PBS three times, cultured with MT2 cells for another 48 h, and finally assessed for luciferase activity (**G**) *p < 0.01.

Our previous study established a Tax-expressing Jurkat cell line (TaxP) and a Tax-negative control line, TaxN, and demonstrated enhanced EGR1 expression in TaxP cells using microarray analysis [5, 25]. To confirm this result, EGR1 expression was measured in Jurkat cells transiently transfected with pCMV-Tax or PmCherry-Tax, as well as in TaxN and TaxP cells. Increased EGR1 mRNA and protein levels were detected in Tax-expressing cells (Figure 1A–1B), suggesting that Tax induced EGR1 expression. Elevated EGR1 was also observed in the HTLV-1-positive cell lines, MT2 and MT4, as compared to the HTLV-1-negative lines, Jurkat and MOLT4 (Figure 1C–1D). EGR1 expression in newly-HTLV-1-infected Jurkat and Hela cells was also examined. Jurkat or Hela cells were co-cultured with different numbers of MT2 cells, which were removed 48 h later. EGR1 mRNA and protein levels, and HTLV-1 Tax protein levels were upregulated in both cell lines (Figure 1E–1F), indicating that early infection also induced EGR1 expression. After co-culturing MT2 cells with Jurkat or Hela cells transfected with EGR1-luc report plasmids, EGR1-mediated transcriptional activity was also increased (Figure 1G), suggesting that HTLV-1 also enhanced EGR1 target gene transcription.

### EGR1 is regulated via SRE elements and an NF-κB binding site

**Figure 2:**
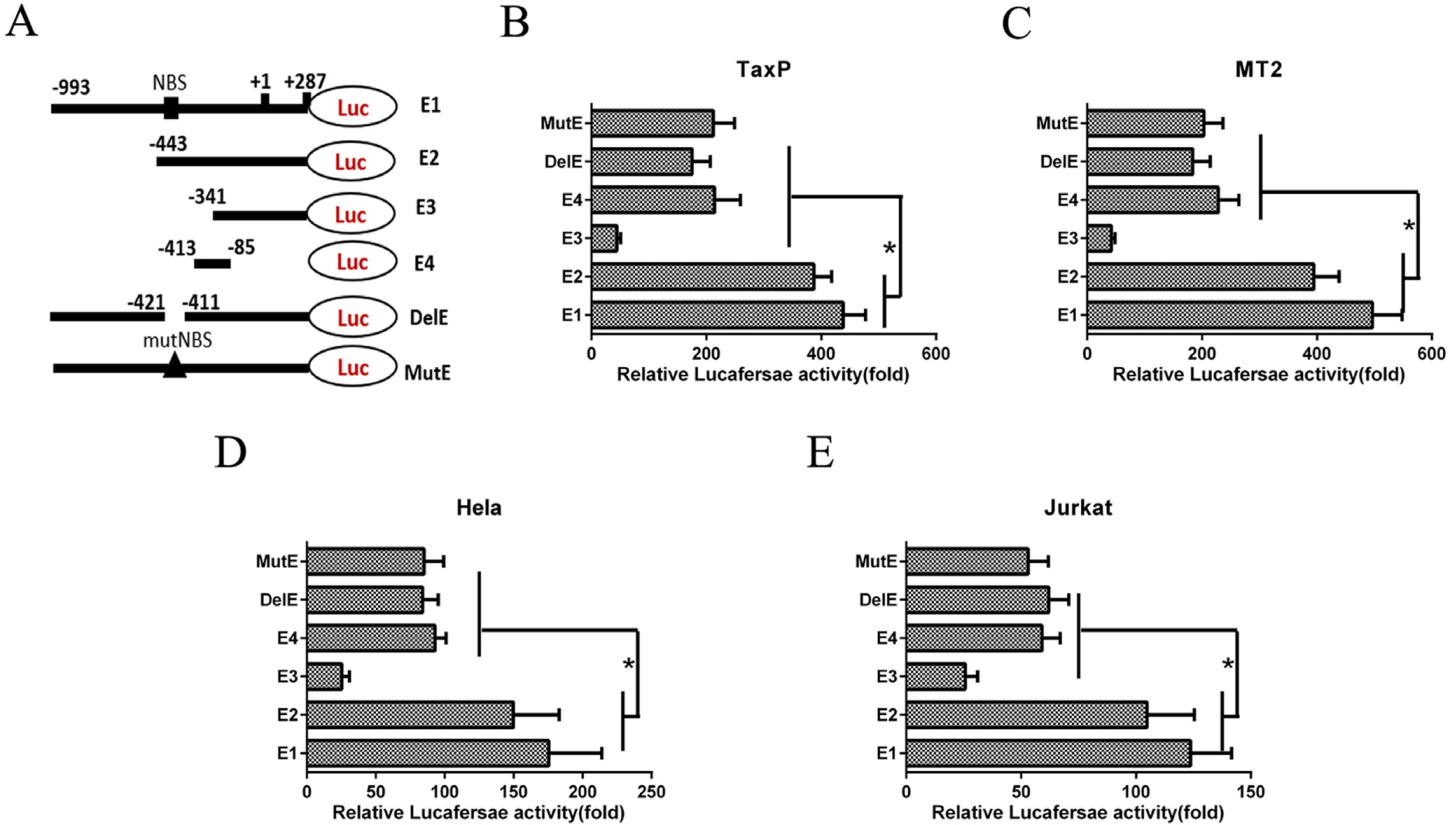
An NF-κB binding site (NBS) in the EGR1 promotor contributes to HTLV-1-induced EGR1 upregulation Schemata showing the luciferase reporter plasmid containing different parts of the EGR1 promotor sequence (**A**) The E1 plasmid contains the full-length EGR1 promotor. E2 and E3 contain –443–+287 and –341–+287, respectively. E4 contains -413–-85. E5 and E6 contain the full-length EGR1 promotor with deleted (DelE) or mutant NBS (MutE), respectively. TaxP (**B**) MT2 (**C**) Hela (**D**) and Jurkat cells (**E**) were transfected with these six luciferase reporter plasmids for 48 h, and luciferase activity was measured. *p < 0.01.

To determine the elements involved in HTLV-1-mediated EGR1 regulation, luciferase reporter plasmids containing different length segments of the EGR1 promoter region (-993–+287, E1) were constructed and transfected into Tax- or HTLV-1-positive cells (Figure 2A). 550 bp and 652 bp fragments were deleted from the promoter region to create E2 and E3 reporter plasmids, respectively (Figure 2A). The E4 plasmid contained five copies of the SRE element, including the CArG box and TCF binding site. Reporter plasmids containing the full-length promotor with a deleted (DelE) or mutant NF-κB binding site (MutE) were also constructed (Figure 2A). Tax-expressing cell lines, TaxP and MT2, were transiently transfected with these reporter plasmids and luciferase activity was measured. Luciferase transcription from the EGR1 promoter region lacking both SRE elements and an NF-κB binding site (E3) was reduced by up to 90% in both cell lines (Figure 2B–2C), indicating a requirement for both of these elements in EGR1 regulation. Transfection with plasmids containing only the SRE element, or with a mutated or deleted NF-κB binding site, reduced transcriptional activity by 50% (Figure 2B–2C). Similar results were observed in Tax-negative Jurkat and Hela cells (Figure 2D–2E), confirming that both the SRE element and NF-κB binding site are essential for regulating EGR1 transcription.

### NF-κB signaling is involved in Tax-induced EGR1 expression

**Figure 3:**
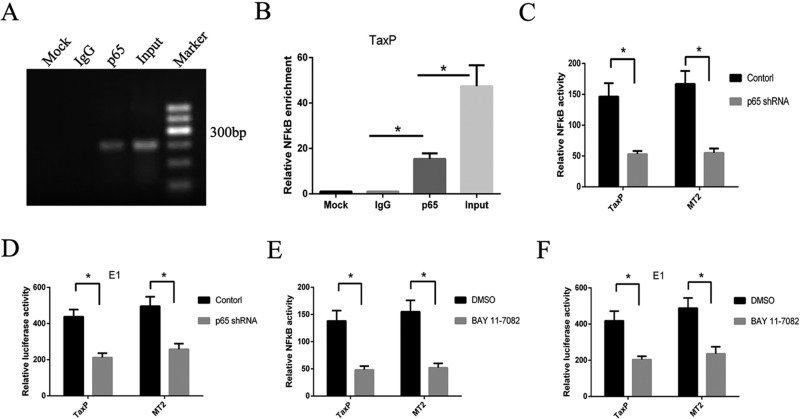
NF-κB pathway inhibition reduces HTLV-1- or Tax-induced EGR1 transcription ChIP assay was performed to determine which EGR1 promotor segments bind to p65 in TaxP cells (**A**). PCR products were subjected to electrophoresis on a 1.2% agarose gel. Results are averaged from three independent ChIP assays (**B**). Error bars show standard deviations. TaxP or MT2 cells were transfected with pNFκB-luc and p65 shRNA, and luciferase activity was measured after 48 h (**C**). TaxP or MT2 cells were transfected with EGR1-luc (E1) and p65 shRNA, and luciferase activity was measured after 48 h (**D**). TaxP or MT2 cells were transfected with pNFκB-luc (**E**) or EGR1-luck (**F**) and treated with Bay 11-7082. After 48 h, luciferase activity was measured. Results are shown as relative expression normalized to the control. *p < 0.01.

The promoting roles of binding sites located in the -413–-85 EGR1 promotor have been reported previously [21], but the contribution of the NF-κB binding site in Tax-induced EGR1 expression has not been studied. Here, we knocked down a key NF-κB protein, p65. In TaxP cells, the p65 protein directly binds the NF-κB binding site in the EGR1 promotor, as measured via CHIP assay (Figure 3A). p65 knockdown in TaxP and MT2 cells using shRNA inhibited NF-κB activation (Figure 3B), and decreased EGR1 transcriptional activity (Figure 3C). Similarly, inhibition of the NF-κB pathway using Bay 11-7082 also inhibited NF-κB activation and EGR1 transcriptional activity (Figure 3D–3E).

### NF-κB activation-defective Tax upregulates EGR1 to a lesser degree than wild-type Tax

**Figure 4:**
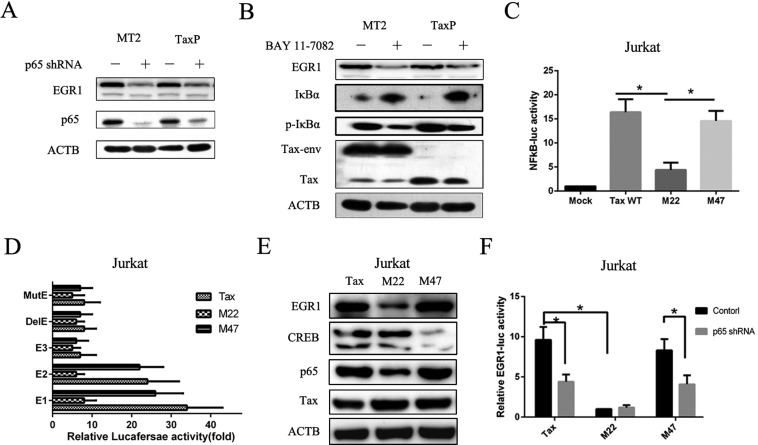
Tax defective for NF-κB activation induces EGR1 expression to a lesser degree compared to wild-type Tax MT2 or TaxP cells were transfected with p65 shRNA for 48 h, and ACTB, EGR1, and p65 were detected by western blot (**A**). MT2 or TaxP cells were treated with or without 5 mM BAY 11-7082 for 48 h, and ACTB, EGR1, and p65, Tax, p-IκB, and IκB were detected by western blot (**B**). Jurkat cells were transfected with pNF-κB-luc and plasmids expressing wild-type Tax, M22 Tax, or M47 Tax for 48 h, and luciferase activity was measured (**C**). Jurkat cells were transfected with the indicated EGR1 promoter-luc plasmids and plasmids expressing wild-type Tax, M22 Tax, or M47 Tax for 48h, and luciferase activity was measured (**D**). Jurkat cells were transfected with plasmids expressing wild-type Tax, M22 Tax, or M47 Tax for 48 h and EGR1, CREB, p65, Tax, and ACTB were detected by western blot E. Jurkat cells were transfected with EGR1-luc plasmids and plasmids expressing wild-type Tax, M22 Tax, or M47 Tax for 48 h, and luciferase activity was measured F. *p < 0.01.

To further confirm the effect of NF-κB activation on EGR1 expression, we assayed protein levels in p65 shRNA-transfected or Bay 11-7082-treated TaxP and MT2 cells. EGR1 was reduced in both cell lines (Figure 4A–4B). Tax mutants M22, which is defective in its ability to activate NF-κB signaling, and M47, which cannot activate CREB [26], were used to examine whether these mutants maintained the ability to induce EGR1 expression. M22 Tax did not activate NF-κB transcriptional activity (Figure 4C and Supplementary Figure 1A) and induced less EGR1 upregulation than wild-type Tax in both Jurkat (Figure 4D–4E) and 293T cells (Supplementary Figure 1B–1C). Moreover, p65 knockdown impaired Tax- and M47 Tax-induced EGR1-mediated transcriptional activity (Figure 4F). These observations confirmed a role for Tax-induced NF-κB activation in enhanced EGR1 expression. M47 Tax had a comparatively reduced effect on EGR1 transcription and expression, suggesting that CREB activation is largely unnecessary.

### Tax directly interacts with and stabilizes the EGR1 protein

**Figure 5:**
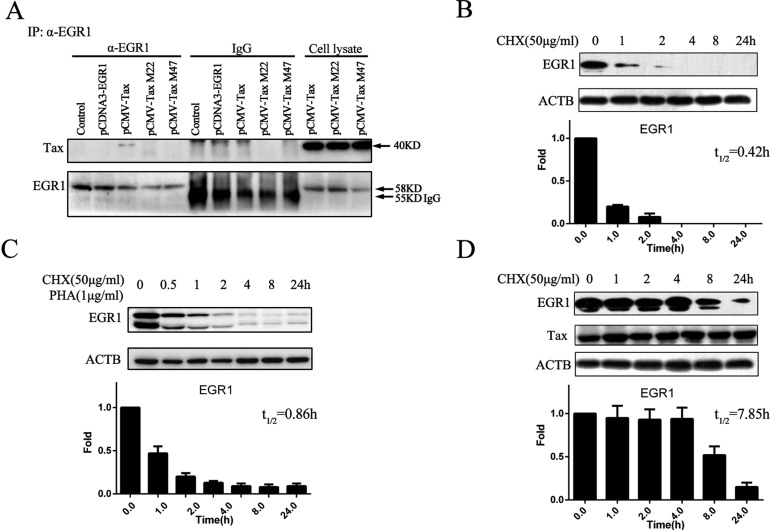
Tax binds to and stabilizes EGR1 293T cells were transfected with pCMV-Tax, pCMV-M22 Tax, or pCMV-M47 Tax and pcDNA3.0-EGR1 for 48 h, and immunoprecipitation was performed to detect interactions between Tax and EGR1 (**A**) Jurkat cells were treated with 50 μg/ml CHX, total protein was extracted at different time points, and EGR1 and ACTB were detected by western blot (**B**) Jurkat cells were treated with 50 μg/ml CHX plus 1 μg/ml PHA, and EGR1 and ACTB were detected by western blot (**C**) Jurkat cells were transfected with pCMV-Tax for 24 h and then treated with 50 μg/ml CHX. EGR1, Tax, and ACTB were detected by western blot (**D**) Protein band pixel densities were quantified from three independent experiments and presented as histograms.

Considering that Tax can affect the functions of various transcriptional factors by binding to them, we investigated whether Tax directly interacted with EGR1. To detect interactions between Tax or Tax mutants and ERG1 using the ERG1 antibody, 293T cells were transfected with Tax- or Tax mutants-expressing plasmids and pcDNA3.0-ERG1 for 48 h. Immunoprecipitation assay results showed that Tax, but not its mutants, co-precipitated with ERG1 (Figure 5A), indicating direct Tax binding to ERG1. In Jurkat cells treated with cycloheximide (CHX), an protein synthesis inhibitor, the EGR1 half-life was 25 min (0.42 h; Figure 5B). This was extended to 0.86 h after stimulation with phytohaemagglutinin (PHA) and CHX (Figure 5C). The EGR1 half-life was 7.85 h in the presence of Tax and CHX (Figure 5D), which suggested enhanced EGR1 stability. However, Tax M47, which cannot bind EGR1, failed to promote EGR1 stability (Supplementary Figure 2).

### EGR1 enhances NF-κB activation via a positive feedback loop

**Figure 6:**
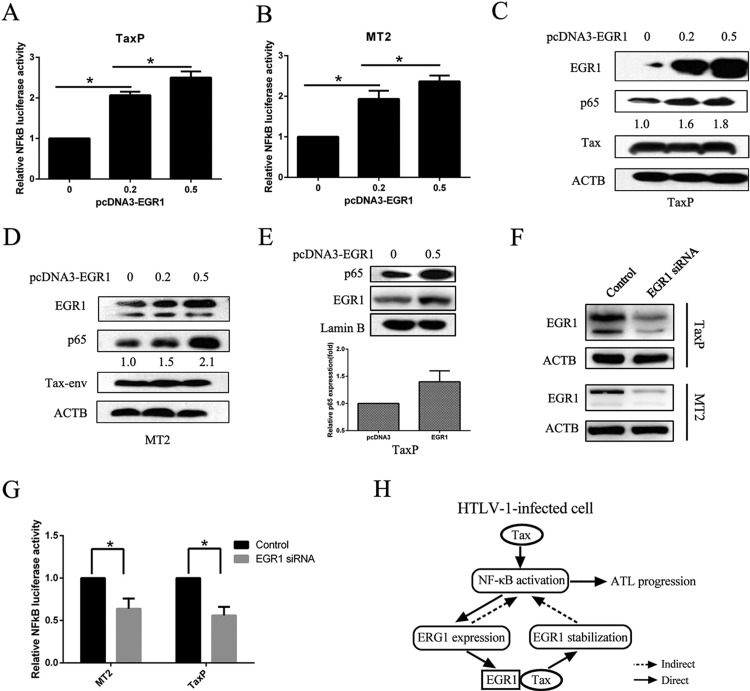
EGR1 overexpression enhances NF-κB activation and p65 nuclear translocation TaxP (**A**) or MT2 cells (**B**) were transfected with pNF-κB-luc and different quantities of pcDNA3.0-EGR1 for 48 h, and luciferase activity was measured. TaxP (**C**) or MT2 (**D**) cells were transfected with different quantities of pcDNA3.0-EGR1 for 48 h, and EGR1, p65, Tax, and ACTB were detected by western blot. TaxP cells were transfected with pcDNA3.0-EGR1 for 24 h, and p65, Lamin B, and EGR1 in the nuclei were analyzed by western blot (**E**) Relative levels of nuclear p65 were analyzed from three individual experiments. TaxP or MT2 cells were transfected with EGR1 siRNA for 48 h and EGR1 was detected by western blot (**F**) MT2 or TaxP cells were transfected with EGR1 siRNA and pNF-κB-luc for 48 h, and luciferase activity was detected (**G**) Schematic showing the positive feedback loop between EGR1 and NF-κB activation in HTLV-1-infected cells (**H**) *p < 0.01.

We examined whether EGR1 affected NF-κB activation. TaxP and MT2 cells were transfected with EGR1-expressing plasmids, and EGR1 upregulation led to enhanced NF-κB signaling (Figure 6A–6B) and dose-dependent p65 upregulation (Figure 6C–6D). Furthermore, EGR1 promoted nuclear translocation of p65 (Figure 6E), and increased NF-κB/DNA binding activity was also observed (Supplementary Figure 3). To confirm the role of EGR1 in NF-κB activation, we knocked down EGR1 in both TaxP and MT2 cells (Figure 6F), and this reduced NF-κB transcriptional activity (Figure 6G).

## DISCUSSION

Persistent activation of the NF-κB pathway induced by HTLV-1 Tax is the main cause of T cell transformation and ATL [27, 28], and Tax alone can induce transcriptional changes in target T cells [27, 29, 30]. We previously found that multiple important transcriptional factors, including Bcl-3, EGR1, and EIF4E2, were upregulated 2-fold by Tax [5, 25]. Our present work demonstrated that Tax upregulates EGR1 expression through the EGR1 promoter's NF-κB binding site, and directly binds the EGR1 protein to promote its stability. As a consequence, elevated EGR1 augments NF-κB activation, which enhances EGR1 transcription in a positive feedback loop (Figure 6H). This feedback loop further facilitates Tax-induced constitutive NF-κB activation in HTLV-1-infected cells, promoting T cell transformation and ATL progression.

EGR1 is a nuclear phosphoprotein that was identified because of its early induction following stimulation of mitogens and differentiation factors [7, 31]. EGR1 expression is low in most normal tissues, with the exception of the brain [32]. EGR1 contains a highly conserved DNA-binding domain composed of three zinc fingers that bind to the prototypic target GC-rich consensus sequence, GCG(G/T)GGGCG [33]. EGR1 can be induced by growth factors, cytokines, and stress signals, such as radiation, injury, ischemia-reperfusion injury or mechanical stress [13]. We found that HTLV-1 infection and the viral protein, Tax, also induce EGR1 expression. Although it is reportedly a tumor suppressor, new evidence suggests that EGR1 promotes prostate cancer progression [34, 35], and might be an effective cancer therapy target [34]. EGR1 in HTLV-1-infected and Tax-expressing cells is upregulated and promotes NF-κB signaling, and may also be a novel anti-ATL therapeutic target.

The EGR1 promoter contains several SREs, an AP-1 binding site, two CREs and an Sp1 consensus sequence [36–39]. An alternative pathway for EGR1 activation has also been discovered, in which NF-κB mediates EGR1 transcription in human skin in response to UV exposure. This study identified a canonical NF-κB binding site in the EGR1 promotor and demonstrated direct binding of p65 to the EGR1 promoter [40]. Our results confirmed that p65 directly binds the EGR1 promoter (-422 bp–-401 bp) to activate EGR1 transcription. Another study identified two functional non-consensus binding sites for the tumor suppressor, p53, in the EGR1 promoter. p53 binding to the EGR1 promoter in response to DNA damage leads to sustained EGR1 expression and efficient apoptosis [41]. In addition, EGR1 binds its own promoter and suppresses its own transcription, initiating a negative feedback loop soon after activation [42]. However, in HTLV-1-infected cells and Tax-expressing cells, EGR1 is consistently upregulated compared to levels in Tax negative cells. We found that this EGR1 dysregulation was caused by Tax-induced persistent activation of the NF-κB pathway, and enhanced EGR1 protein stability via direct Tax binding.

Fujii, *et al*. identified the CArG boxes as Tax-responsive cis-acting elements for regulating the cellular immediate early genes, c-fos, egr-1, and egr-2 [43]. This might represent a mechanism through which Tax alters infected cell growth, since Tax activates CArG-mediated transcription through interaction with a CArG-binding factor, p67SRF, independent of mitogenic signals [43]. In our study, absence of -993 bp–-443 bp in the EGR1 promotor (E2) only slightly reduced EGR1 transcriptional activity, suggesting that the relevant binding sites are not localized in this area. Binding sites located in -443 bp–-341 bp, which contains three CArG boxes (-413 bp –-405 bp, -376 bp–367 bp, and -360 bp–-351 bp), two TCF binding sites (-400 bp, -384 bp), and one NF-κB binding site (-422 bp–-401 bp), play crucial roles in EGR1 transcription, since absence of this area reduced transcriptional activity most significantly. In addition, the NF-κB inhibitor, BAY 11–7082, p65 knockdown, or Tax defective for NF-κB activation each reduced Tax-mediated EGR1 upregulation, further confirming the importance of NF-κB activation in EGR1 expression. Moreover, Tax binds to and stabilizes EGR1, revealing a novel mechanism for elevated EGR1 in Tax-expressing cells.

NF-κB and CREB proteins play central roles in HTLV-1-infected T lymphocyte activation [44]. Our results showed that higher EGR1 levels in Tax-positive cells further increased p65 expression and promoted its nuclear translocation, leading to enhanced NF-κB binding activity, which favors T cell transformation and EGR1 upregulation. Similar results were observed in cells expressing the HIV-encoded protein, Tat, suggesting that Tat-induced EGR1 interacts with p65 *in vitro* and regulates NF-κB transcriptional activity *in vivo* [24, 45]. The NF-κB binding site is reportedly important for EGR1-mediated IL-8 upregulation, and EGR1 knockdown inhibits IL-8 production and IL-8-mediated prostate cancer cell invasion. This inhibition appears to be dependent on suppressing EGR1/NF-κB synergy [46, 47], indicating a close relationship and cross-talk between EGR1 and the NF-κB pathway.

In summary, our results demonstrate a positive feedback loop between EGR1 expression and NF-κB activation in HTLV-1-infected and Tax-expressing cells. Elevated EGR1 likely contributes to ATL progression by upregulating pro-transformation genes and facilitating constitutive NF-κB activation. Since both EGR1 and the NF-κB pathway play crucial roles in T cell transformation and ATL progression, ERG1 blockade in HTLV-1-infected patients may delay or prevent development of ATL.

## MATERIALS AND METHODS

### Cell culture

T cell lines (Jurkat and MOLT4), HTLV-1-infected T cell lines (MT2 and MT4), Hela cells, and 293T cells were cultured at 37°C in RPMI 1640 medium supplemented with 10% heat-inactivated fetal calf serum, penicillin (100 units/ml), and streptomycin (100 mg/ml). TaxN and TaxP cells were cultured in complete medium supplemented with the antibiotic, G418 (300 μg/ml, Beyotime Biotechnology, Shanghai, China).

### Antibodies and reagents

Anti-EGR1 (22008-1-AP) rabbit polyclonal antibody (pAb), anti-Lamin B (66095-1-Ig), and anti-tubulin (66031-1-Ig) were purchased from ProteinTech (Rosemont, IL, USA). Antibodies against IκBα (4814S), p-IκBα (Ser32) (2859S), and p-p65 (8242S) were purchased from Cell Signaling Technology (Danvers, MA, USA). Anti-p65 rabbit pAb (ab7970) and anti-Tax mouse monoclonal antibody (mAb, sc-57872) were bought from Abcam (Cambridge, MA, USA) and Santa Cruz Biotechnology (Santa Cruz, CA, USA), respectively. The anti-β-actin mouse mAb (60008-1-Ig), as well as the following immunoglobulin G (IgG) reagents: horseradish peroxidase-linked goat anti-mouse (00001-1) and goat anti-rabbit (00001-2), were purchased from ProteinTech. The NF-κB inhibitor, Bay 11-7082 (B5556), and phytohemagglutinin (PHA; L8902) were purchased from Sigma (St Louis, MO, USA). 5-(and-6)-Carboxy fluorescein diacetate, succinimidyl ester (CFSE) (C1157), EGR1 siRNA (1299001), and Lipofectamine 2000 (11668-019) were purchased from Invitrogen (Carlsbad, CA, USA). Cycloheximide (CHX; C8030) was purchased from Solarbio (Beijing, China).

### Quantitative real-time RT-PCR

RNA was extracted from cells using the Trizol reagent (15596026, Invitrogen) and RNeasy mini kit (74104, Qiagen, Hilden, Germany). mRNA was quantified via real-time RT-PCR using the GoTaq^®^ qRCR Master Mix (A6002, Promega, Madison, WI, USA) according to the manufacturer's instructions. The relative amount of each gene compared to the ACTB internal control, and the fold stimulation were calculated using the 2^–ΔΔCT^ method. *EGR1* was amplified using specific primers (sense: CCCCGACTACCTGTTTCCAC and anti-sense: TGGGTTTGATGAGCTGGGAC). Results are the average of three separate experiments.

### Western blot

Total protein was extracted from cells using RIPA buffer and separated in 12% SDS-PAGE gel. Proteins were transferred to PVDF membranes (Millipore Corporation, Bedford, MA, USA) and incubated with primary antibodies followed by anti-rabbit or anti-mouse IgG secondary antibodies conjugated to horseradish peroxidase. Bands were visualized and imaged using enhanced chemiluminescence reagent (P0018, Beyotime Biotechnology).

### Plasmids construction

The 1397bp DNA fragment of the EGR1 promotor was amplified from genomic DNA using specific primers (forward 5′-CACCCAGGCCTCTCTTGGGGC AATCA-3′, reverse 5′-TGTCCATGGTGGGCGAGT GA-3′). This fragment was cloned into the pMD18-T vector (TaKaRa). E1, E2 and E3 fragments were PCR amplified using pMD18-T-EGR1 as a template with the following specific primers: forward E1 5′-GGGGTACCAGCTTCC CCACTTCGGTCCCC-3′, -993–+287 bp; E2 5′-GGGGTA CCGAGGGAGCAACCAGCTGCGA-3′, -443–+287 bp; E3 5′-GGGGTACCGTGCAGGATGGAGGTGCCGG-3′, -341–+287 bp; E4 5′-GGGGTACCGCCATATAAGGAG CAGGAAGG-3′, -414–-85 bp; and reverse 5′-CCCAAG CTTCCTGGACGAGCAGGCTGGAGA-3′ (underlining indicates restriction enzyme clone sites. PCR fragments were digested with Hind III and Kpn I, and were cloned into the pGL3-Basic-luc vector. The deleted DelE (del-NF-κB) and mutated MutE (mut-NF-κB) constructs were created according to the Quick change protocol (200518, TaKaRa, Shiga, Japan) using the following primers: DelE 5′-TTGGAACCAGGGAGGAGGGAGGGAGCGGAAA TGCCATATAAGG AGCAGGA-3′; MutE 5′-GGAGCAA CCAGCTGCGACCCGACTAGTCCATAT AAGGAGCA GGAAGGATC-3′. Total mRNA was extracted from MT2 cells using the RNeasy mini kit. The 1632bp EGR1 complete CDS was amplified via RT-PCR using specific primers (forward 5′-CCCAAAGCTTGGGA TGGCCGCGGCCAAGGCC-3′, reverse 5′-GGAATTCC GGGTTTAGCAAATTTCAATTGTCCTGGGAG-3′). This fragment was treated with HindIII and EcoRI and then cloned into the pcDNA3.0 vector (Invitrogen). pNF-κB-luc plasmids were kindly provided by Haojiang Luan (National Institute of Mental Health, NIH), and pCMV-Bam-Tax and pCMV-Bam plasmids were kindly provided by Shoji Yamaoka (Tokyo Medical and Dental University). To construct a plasmid expressing full-length Tax with an RFP tag, the Tax CDS was subcloned from pCMV-Bam-Tax into the Bgl II and Hind III sites of the pmCherry-C1 vector. pCMV-M22 Tax and pCMV-M47 Tax plasmids were kindly provided by Edward Harhaj (The University of Miami). p65 shRNA (sc-29410-SH) was purchased from Santa Cruz. EGR1 luciferase reporter plasmid containing EGR1 binding sites was purchased from Yeasen Biotech (Shanghai, China).

### Luciferase reporter assay

Cells were transfected with pNF-κB-luc, EGR1-luc, or different EGR1 promoter -luc plasmids, and enzymatic activity was examined in cell extracts using the Luciferase Assay System (Promega, E1500) and a 20/20n Luminometer (Turner BioSystems) according to the manufacturer's instructions. All reporter assays were performed in triplicate and repeated at least three times.

### siRNA transfection

MT2 or TaxP cells were seeded into 24-well plates and transfected with 50 nM EGR1 siRNA (5′-GAUC UCUGACCCGUUCGGAUCCUUU-3′, 5′-AAAGGAU CCGAACGGGUCAGAGAUC-3′) and/or pNF-κB-luc using Lipofectamine 2000 (Invitrogen). After 48 h, cells were collected for EGR1 detection or for luciferase activity measurement.

### Chromatin immunoprecipitation assay

According to the protocol recommended by Upstate Biotechnology, TaxP cells (1 × 10^7^) were collected, fixed with 1% formaldehyde for 10 min to crosslink proteins to DNA, and then quenched with 10× glycine for 5 min. After washing with ice-cold PBS, cells were suspended in SDS lysis buffer. Chromatin was then sheared to a manageable size with 5 sets of 10 s pulses on wet ice using a 100-watt model. ChIPs were performed using the chromatin immunoprecipitation Kit (17–295, Millpore). Sheared chromatin was immunoprecipitated using a specific antibody directed against p65, and a nonimmune serum as a negative control. Then, protein/DNA complexes were reversed by incubating samples at 65°C overnight. Associated DNA was eluted and purified according to the manufacturer's instructions. Captured EGR1 regulatory sequences were identified via PCR analysis using the following primers: 5′- CTCCCGGCTTGGAACCAG-3′ and 5′- CC TTCTTCCCTCCTCCCAGA -3′; 179 bp (-476 bp–-298 bp). One microliter of precipitated and purified DNA was subjected to standard PCR, and DNA fragment sizes were analyzed using 2% agarose gel electrophoresis. Detection of specific DNA sequences was performed via qPCR analysis of ChIPed samples using the GoTaq^®^ qRCR Master Mix (Promega). The EGR1 promotor sites bound by p65 protein were determined using qPCR analysis. The CT value at each p65-targeted site was normalized to the negative control, which was set to 1.

### Co-immunoprecipitation

293T cells were co-transfected with pCMV-Tax, pCMV-M22Tax, or pCMV-M47 Tax and/or pcDNA3.0-EGR1 for 48 h. Cells were harvested and interactions between EGR1 and Tax were detected using immune co-precipitation (Nuclear Complex Co-IP Kit; 54001, Active Motif). An EGR1 monoclonal antibody (ab55160, Abcam) was used to capture EGR1 protein and the target protein, Tax, was detected via western blot.

### Statistical analysis

Statistical significance for the luciferase reporter and qRT-PCR assays was determined using the Student's *t-test* or one-way ANOVA. *P <* 0.05 was regarded as statistically significant.

## SUPPLEMENTARY MATERIALS FIGURES



## References

[1] Poiesz BJ, Ruscetti FW, Gazdar AF, Bunn PA, Minna JD, Gallo RC (1980). Detection and isolation of type C retrovirus particles from fresh and cultured lymphocytes of a patient with cutaneous T-cell lymphoma. Proc Natl Acad Sci USA.

[2] Hinuma Y, Nagata K, Hanaoka M, Nakai M, Matsumoto T, Kinoshita KI, Shirakawa S, Miyoshi I (1981). Adult T-cell leukemia: antigen in an ATL cell line and detection of antibodies to the antigen in human sera. Proc Natl Acad Sci USA.

[3] Matsuoka M, Jeang KT (2007). Human T-cell leukaemia virus type 1 (HTLV-1) infectivity and cellular transformation. Nat Rev Cancer.

[4] Higuchi M, Fujii M (2009). Distinct functions of HTLV-1 Tax1 from HTLV-2 Tax2 contribute key roles to viral pathogenesis. Retrovirology.

[5] Wang J, Niu Z, Shi Y, Gao C, Wang X, Han J, Li J, Gao Z, Zhu X, Song X, Qin Z, Wang H (2013). Bcl-3, induced by Tax and HTLV-1, inhibits NF-kappaB activation and promotes autophagy. Cell Signal.

[6] Pagel JI, Deindl E (2011). Early growth response 1—a transcription factor in the crossfire of signal transduction cascades. Indian J Biochem Biophys.

[7] Zwang Y, Oren M, Yarden Y (2012). Consistency test of the cell cycle: roles for p53 and EGR1. Cancer Res.

[8] Virolle T, Krones-Herzig A, Baron V, De Gregorio G, Adamson ED, Mercola D (2003). Egr1 promotes growth and survival of prostate cancer cells. Identification of novel Egr1 target genes. J Biol Chem.

[9] McMahon SB, Monroe JG (1996). The role of early growth response gene 1 (egr-1) in regulation of the immune response. J Leukoc Biol.

[10] Chasseigneaux S, Dinc L, Rose C, Chabret C, Coulpier F, Topilko P, Mauger G, Allinquant B (2011). Secreted amyloid precursor protein beta and secreted amyloid precursor protein alpha induce axon outgrowth *in vitro* through Egr1 signaling pathway. PLoS One.

[11] Guerquin MJ, Charvet B, Nourissat G, Havis E, Ronsin O, Bonnin MA, Ruggiu M, Olivera-Martinez I, Robert N, Lu Y, Kadler KE, Baumberger T, Doursounian L (2013). Transcription factor EGR1 directs tendon differentiation and promotes tendon repair. J Clin Invest.

[12] Woodman I (2013). Development, EGR1 is a key factor in tendon development and healing. Nat Rev Rheumatol.

[13] Feng Y, Desjardins CA, Cooper O, Kontor A, Nocco SE, Naya FJ (2015). EGR1 Functions as a Potent Repressor of MEF2 Transcriptional Activity. PLoS One.

[14] Perez-Castillo A, Pipaon C, Garcia I, Alemany S (1993). NGFI-A gene expression is necessary for T lymphocyte proliferation. J Biol Chem.

[15] Verduci L, Azzalin G, Gioiosa S, Carissimi C, Laudadio I, Fulci V, Macino G (2015). microRNA-181a enhances cell proliferation in acute lymphoblastic leukemia by targeting EGR1. Leuk Res.

[16] Wang J, Li Y, Liu Y, Gong S, Fang F, Wang Z (2015). Overexpression of truncated AIF regulated by Egr1 promoter radiation-induced apoptosis on MCF-7 cells. Radiat Environ Biophys.

[17] Sharma A, Kumar M, Aich J, Hariharan M, Brahmachari SK, Agrawal A, Ghosh B (2009). Posttranscriptional regulation of interleukin-10 expression by hsa-miR-106a. Proc Natl Acad Sci USA.

[18] Li H, Li J, Jia S, Wu M, An J, Zheng Q, Zhang W, Lu D (2015). miR675 upregulates long noncoding RNA H19 through activating EGR1 in human liver cancer. Oncotarget.

[19] Contreras JR, Palanichamy JK, Tran TM, Fernando TR, Rodriguez-Malave NI, Goswami N, Arboleda VA, Casero D, Rao DS (2015). MicroRNA-146a modulates B-cell oncogenesis by regulating Egr1. Oncotarget.

[20] Ramadas N, Rajaraman B, Kuppuswamy AA, Vedantham S (2014). Early growth response-1 (EGR-1) - a key player in myocardial cell injury. Cardiovasc Hematol Agents Med Chem.

[21] Shuh M, Derse D (2000). Ternary complex factors and cofactors are essential for human T-cell leukemia virus type 1 tax transactivation of the serum response element. J Virol.

[22] Nishitsuji H, Sawada L, Sugiyama R, Takaku H (2015). ZNF10 inhibits HIV-1 LTR activity through interaction with NF-kappaB and Sp1 binding motifs. FEBS Lett.

[23] Chapman NR, Perkins ND (2000). Inhibition of the RelA(p65) NF-kappaB subunit by Egr-1. J Biol Chem.

[24] Dron M, Hameau L, Benboudjema L, Guymarho J, Cajean-Feroldi C, Rizza P, Godard C, Jasmin C, Tovey MG, Lang MC (1999). Cloning of a long HIV-1 readthrough transcript and detection of an increased level of early growth response protein-1 (Egr-1) mRNA in chronically infected U937 cells. Arch Virol.

[25] Wang J, Li J, Huang Y, Song X, Niu Z, Gao Z, Wang H (2013). Bcl-3 suppresses Tax-induced NF-kappaB activation through p65 nuclear translocation blockage in HTLV-1-infected cells. Int J Oncol.

[26] Robek MD, Ratner L (1999). Immortalization of CD4(+) and CD8(+) T lymphocytes by human T-cell leukemia virus type 1 Tax mutants expressed in a functional molecular clone. J Virol.

[27] Lavorgna A, Harhaj EW (2014). Regulation of HTLV-1 tax stability, cellular trafficking and NF-kappaB activation by the ubiquitin-proteasome pathway. Viruses.

[28] Gross C, Thoma-Kress AK (2016). Molecular Mechanisms of HTLV-1 Cell-to-Cell Transmission. Viruses.

[29] Boxus M, Twizere JC, Legros S, Dewulf JF, Kettmann R, Willems L (2008). The HTLV-1 Tax interactome. Retrovirology.

[30] Chlichlia K, Khazaie K (2010). HTLV-1 Tax: Linking transformation, DNA damage and apoptotic T-cell death. Chem Biol Interact.

[31] Milbrandt J (1987). A nerve growth factor-induced gene encodes a possible transcriptional regulatory factor. Science.

[32] Veyrac A, Besnard A, Caboche J, Davis S, Laroche S (2014). The transcription factor Zif268/Egr1, brain plasticity, and memory. Prog Mol Biol Transl Sci.

[33] O’Donovan KJ, Tourtellotte WG, Millbrandt J, Baraban JM (1999). The EGR family of transcription-regulatory factors: progress at the interface of molecular and systems neuroscience. Trends Neurosci.

[34] Gitenay D, Baron VT (2009). Is EGR1 a potential target for prostate cancer therapy?. Future Oncol.

[35] Abdulkadir SA (2005). Mechanisms of prostate tumorigenesis: roles for transcription factors Nkx3.1 and Egr1. Ann N Y Acad Sci.

[36] Tsai-Morris CH, Cao XM, Sukhatme VP (1988). 5′ flanking sequence and genomic structure of Egr-1, a murine mitogen inducible zinc finger encoding gene. Nucleic Acids Res.

[37] Schwachtgen JL, Campbell CJ, Braddock M (2000). Full promoter sequence of human early growth response factor-1 (Egr-1): demonstration of a fifth functional serum response element. DNA Seq.

[38] Sakamoto KM, Bardeleben C, Yates KE, Raines MA, Golde DW, Gasson JC (1991). 5′ upstream sequence and genomic structure of the human primary response gene, EGR-1/TIS8. Oncogene.

[39] Christy B, Nathans D (1989). Functional serum response elements upstream of the growth factor-inducible gene zif268. Mol Cell Biol.

[40] Thyss R, Virolle V, Imbert V, Peyron JF, Aberdam D, Virolle T (2005). NF-kappaB/Egr-1/Gadd45 are sequentially activated upon UVB irradiation to mediate epidermal cell death. EMBO J.

[41] Yu J, Baron V, Mercola D, Mustelin T, Adamson ED (2007). A network of p73, p53 and Egr1 is required for efficient apoptosis in tumor cells. Cell Death Differ.

[42] Cao X, Mahendran R, Guy GR, Tan YH (1993). Detection and characterization of cellular EGR-1 binding to its recognition site. J Biol Chem.

[43] Fujii M, Tsuchiya H, Chuhjo T, Akizawa T, Seiki M (1992). Interaction of HTLV-1 Tax1 with p67SRF causes the aberrant induction of cellular immediate early genes through CArG boxes. Genes Dev.

[44] Kato K, Akashi K (2015). Recent Advances in Therapeutic Approaches for Adult T-cell Leukemia/Lymphoma. Viruses.

[45] Fan Y, Zou W, Green LA, Kim BO, He JJ (2011). Activation of Egr-1 expression in astrocytes by HIV-1 Tat: new insights into astrocyte-mediated Tat neurotoxicity. J Neuroimmune Pharmacol.

[46] Ma J, Ren Z, Ma Y, Xu L, Zhao Y, Zheng C, Fang Y, Xue T, Sun B, Xiao W (2009). Targeted knockdown of EGR-1 inhibits IL-8 production and IL-8-mediated invasion of prostate cancer cells through suppressing EGR-1/NF-kappaB synergy. J Biol Chem.

[47] Singha B, Gatla HR, Manna S, Chang TP, Sanacora S, Poltoratsky V, Vancura A, Vancurova I (2014). Proteasome inhibition increases recruitment of IkappaB kinase beta (IKKbeta), S536P-p65, and transcription factor EGR1 to interleukin-8 (IL-8) promoter, resulting in increased IL-8 production in ovarian cancer cells. J Biol Chem.

