# Supplementary material for: HTLV-1 Tax upregulates early growth response protein 1 through nuclear factor-κB signaling
Source: Oncotarget. 2017 May 8;8(31):51123–33. doi: 10.18632/oncotarget.17699 (PMC5584236; doi:10.18632/oncotarget.17699)
Supplement: Supplementary file 1 [file oncotarget-08-51123-s001.pdf]

HTLV-1 Tax upregulates early growth response protein 1 through nuclear factor-κB signaling

Supplementary Materials

Nuclear extracts and electrophoretic mobility shift assay

Nuclear were extracted from MT2 cells using Nuclear Extract Kit (40010, Active Motif, Carlsbad, CA, USA). DNA binding reactions were performed in a volume of 25 ml of 10 mg nuclear protein in the appropriate binding buffer (NF-κB 5X buffer: Tris 50 mM pH 7.5, NaCl 500 mM, EDTA 5 mM, glycerol 20%, salmon sperm DNA 0.4 mg/ml, DTT 5 mM; EGR1 5× buffer: HEPES 50 mM pH 7.9, glycerol 50%, DTT 5mM, KCl 250 mM, MgCl2 12.5 mM, salmon sperm DNA 0.4 mg/ml). In all, 200 000 cpm of ^{32}P-radiolabeled double-stranded DNA probe (NF-κB-probe, GS056, Beyotime) were used per reaction. The binding reaction was pre-incubated with competitors (homologous cold probe, mutated cold probe and antibodies) for 15 min at 4°C and then incubated with the radiolabeled probe for 20 min at room-temperature. DNA/protein complexes were resolved on 5% poly-acrylamide gels and detected by autoradiography.

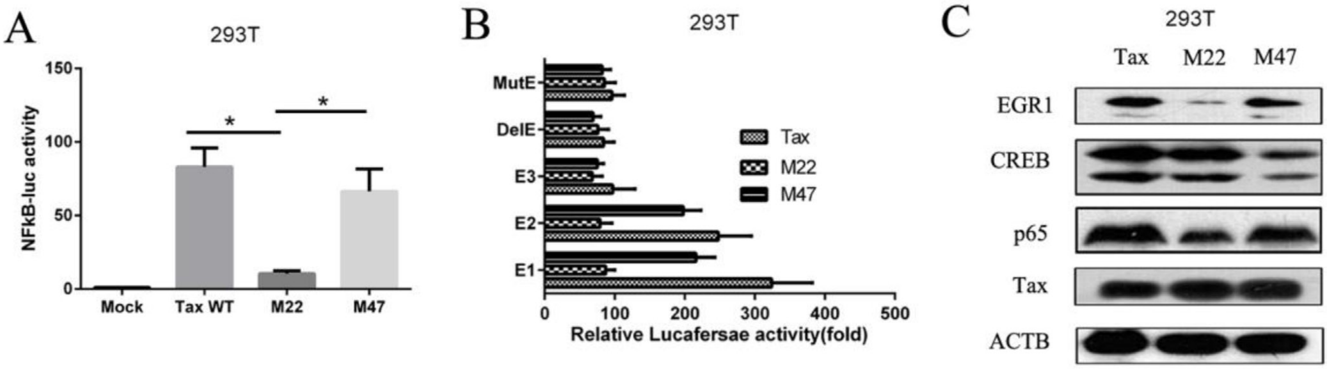

Supplemental Figure 1: Tax defective NF-κB activation induces less EGR1 expression. (**A**) 293T cells were transfected with pNFκB-luc and plasmids expressing wild-type Tax, M22 Tax, or M47 Tax for 48h, following which, the luciferase activity was measured. (**B**) 293T cells were transfected with indicated types of EGR1 promoter-luc plasmids and plasmids expressing wild-type Tax, M22 Tax, or M47 Tax for 48 h, following which, the luciferase activity was measured. (**C**) 293T cells were transfected with plasmids expressing wild-type Tax, M22 Tax, or M47 Tax for 48 h and the proteins EGR1, CREB, p65, Tax, and ACTB were detected by Western blot.

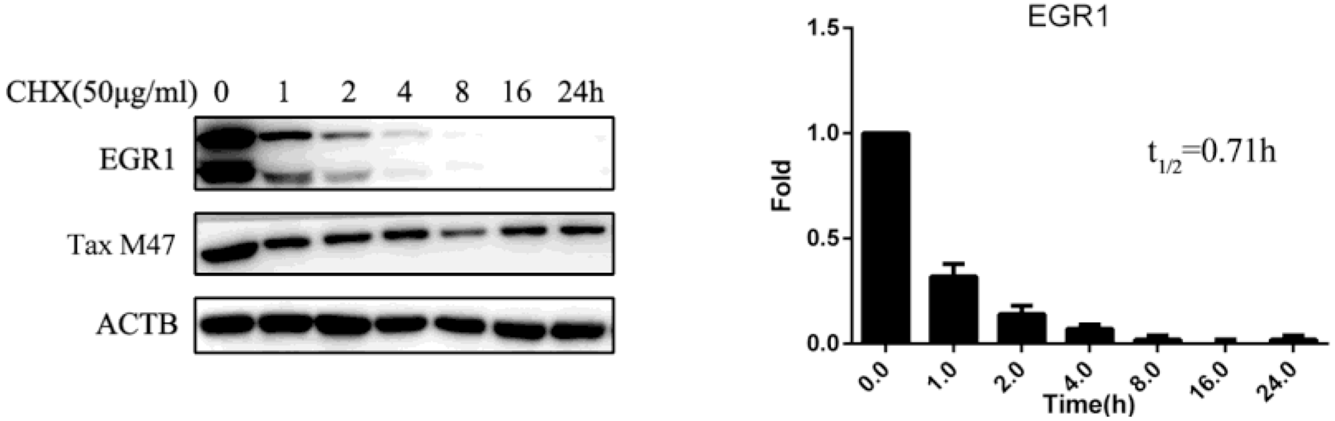

Supplemental Figure 2: M47 Tax cannot induce EGR1 stability. Jurkat cells were transfected with pCMV-Tax M47 for 24 h and then treated with 50 μg/ml CHX for the indicated times. The expression of EGR1, Tax, and ACTB in these cells was detected by Western blot. The pixel densities of the proteins were quantified from three independent experiments and presented as histograms.

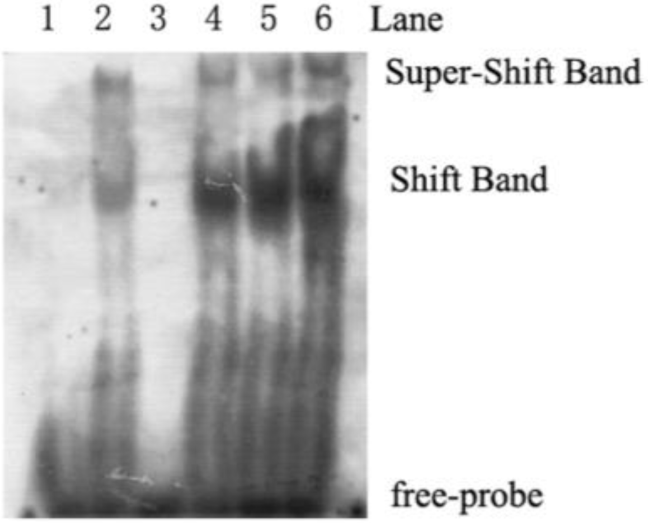

Supplemental Figure 3: EGR1 increases the NF-κB /DNA binding activity. TaxP cells were transfected with pcDNA 3.0 or pcDNA3.0-EGR1 for 48 h and nuclear extracts obtained from these cells were used to perform EMSA assay. Nuclear extracts obtained from TaxP cells without transfection (lane 2 and 3) were incubated with ^{32}P-labeled NF-κB oligonucleotide probe in the presence of a 100-fold excess of unlabeled NF-κB oligonucleotide (lane 3) or the presence of a 100-fold excess of unlabeled NF-κB mutated cold probe (lane 2). Nuclear extracts obtained from TaxP cells (lane 4) or TaxP cells transfected with pcDNA3.0 (lane 5) or pcDNA3.0-EGR1 (lane6) were incubated with ^{32}P-labeled NF-κB oligonucleotide probe. The anti-p65 antibody was added into all these groups (lane 2–6). A reaction without the nuclear extract was performed as a mock (lane 1).
